# Twenty-four hour Blood Pressure in Obese Patients with Moderate-to-Severe Obstructive Sleep Apnea

**DOI:** 10.5935/abc.20170130

**Published:** 2017-10

**Authors:** Claudia M. Correa, Ronaldo A. Gismondi, Ana Rosa Cunha, Mario F. Neves, Wille Oigman

**Affiliations:** 1 Hospital Universitário Pedro Ernesto - Universidade Estadual do Rio de Janeiro (UERJ), Rio de Janeiro, RJ - Brazil; 2 Universidade Federal Fluminense (UFF), Niterói, RJ - Brazil

**Keywords:** Blood Pressure, Sleep Apnea, Obstructive, Hypertension, Blood Pressure Monitoring, Ambulatory

## Abstract

**Background:**

Obesity, systemic arterial hypertension (SAH) and obstructive sleep apnea (OSA) are closely related. Up to 70% of patients with OSA may be asymptomatic, and there is evidence that these patients have cardiovascular disease, especially nocturnal SAH.

**Objectives:**

The aim of this study was to evaluate 24-hour blood pressure circadian variation in asymptomatic, obese individuals with moderate-to-severe OSA and compare it with that in individuals with mild OSA or without OSA.

**Methods:**

Eighty-six obese subjects aged between 30 and 55 years (BMI 30-39 kg/m^2^), with casual blood pressure < 140/90 mmHg and without comorbidities were recruited. Eighty-one patients underwent clinical and anthropometric assessment, ambulatory blood pressure monitoring (ABPM), and Watch-PAT. Participants were divided into two groups, based on the apnea-hypopnea index (AHI): group 1, with AHI < 15 events/hour, and group 2 with AHI ≥ 15 events/hour.

**Results:**

Compared with group 1, group 2 had higher neck circumference and waist-hip circumference (40.5 ± 3.2 cm vs. 38.0 ± 3.7 cm, p = 0.002, and 0.94 ± 0.05 vs. 0.89 ± 0.05, p = 0.001, respectively), higher systolic and diastolic blood pressure measured by the 24-h ABPM (122 ± 6 vs 118 ± 8 mmHg, p = 0.014, and 78 ± 6 vs 73 ± 7 mmHg, p = 0.008, respectively), and higher nocturnal diastolic pressure load (44,6 ± 25,9% vs 31,3 ± 27,3%, p = 0,041). Moreover, there was a positive correlation between nocturnal diastolic blood pressure and AHI (r = 0.43, p < 0.05).

**Conclusions:**

Asymptomatic obese subjects with moderate-to-severe OSA have higher systolic and diastolic blood pressure at 24 hours compared with those with absent / mild OSA, despite normal casual blood pressure between the groups. These results indicate that ABPM may be useful in the evaluation of asymptomatic obese patients with moderate-to-severe OSA.

## Introduction

Obstructive sleep apnea (OSA) is the most common sleep respiratory disorder^1,2^ characterized by repetitive collapse of the upper airway that causes pauses in respiration and intermittent hypoxia.^2^ During these nocturnal episodes of obstruction, there is an increase in sympathetic tonus and in the release of vasoactive substances, leading to increased risk of cardiovascular injury.^3^

A recent systematic review estimated that the prevalence of OSA is higher among men, and ranges from 9 and 38% in the general population.^4^ In the study by Tufik et al.,^5^ performed in the city of Sao Paulo, Brazil, OSA was observed in 32,8% of the participants. However, according to the classic study Sleep Health Study, many OSA patients are asymptomatic, as 70% of patients with mild apnea and 9% of those with severe apnea were asymptomatic.^6^

OSA is mostly related to obesity and systemic arterial hypertension (SAH).^2^ Obese patients have higher prevalence of OSA and SAH^7^ and the association of obesity with SAH may cause target-organ injury and cardiovascular events.^8,9^ Studies have demonstrated that OSA patients have less nocturnal blood pressure (BP) dipping and nocturnal hypertension.^10,11^ Most of these reports have included hypertensive patients with previous diagnosis of OSA. A previous study suggested a higher prevalence of masked hypertension (MH) in patients with OSA.^8^ However, little is known about the 24-hour BP behavior in obese individuals with OSA and normal casual BP. The aim of this study was to assess 24-hour BP circadian variation in obese, asymptomatic subjects with moderate-to-severe OSA compared with subjects with mild OSA or without OSA.

## Methods

### Subjects

In the period from January to December 2014, individuals attending the internal medicine outpatient clinic of Piquet Carneiro Polyclinic of Rio de Janeiro State University (UERJ) were invited to participate in the study. Inclusion criteria were age between 30 and 55 years, body mass index (BMI) between 30 and 39.9 kg/m^2^, normal casual BP (BP < 140/90 mmHg), and absence of comorbidities, and regular follow-up. Exclusion criteria were history of arterial hypertension or treatment for this condition, diabetes mellitus, pulmonary disease, Parkinson disease, previous therapy with continuous positive airway pressure (CPAP), and previous diagnosis of OSA.

The study was approved by the local ethics committee (*Plataforma Brasil/CAAE*: 03489612.1.0000.52590), and informed consent was obtained from all participants.

### Study design

This was a cross sectional, observational study. At the first visit, patients underwent clinical, anthropometrical, and laboratory assessments, and diagnostic test for OSA. At the second visit, ambulatory blood pressure monitoring (ABPM) was performed, with a maximum interval of one-week between the visits.

### Anthropometric measures

Body weight was measured with participants standing at the center of the platform, wearing light clothes and barefoot, using a Filizola® digital scale with maximum capacity of 180 kg.^12^ Height was measured using the vertical rod attached to the same scale, with patients standing straight and heels together. Body mass index (BMI) was then calculated, by dividing body weight (kg) by height (m^2^). Circumferences were measured using an inextensible, graduated measuring tape.^13^ Neck circumference (NC) was at the level of cricoid cartilage; waist circumference (WC) was measured at the midpoint between the lower rib and the iliac crest at the end of expiratory phase of respiration. Hip circumference was measured at the femoral trochanters.^14^ All measures were taken in cm and at the nearest 0.5 cm.

### Blood pressure

Casual BP was measured using an electronic device (HEM-705CP, Omron Healthcare Inc., Lake Forest, IL, USA) and a cuff with adequate size, according to patient’s arm circumference, following the Brazilian Guidelines on Hypertension.^15^ Before the measurement, participants remained seated for 30 minutes and refrained from coffee and smoking. Three measurements were taken with a one-minute interval between them, and the mean of these three measurements was defined as casual BP.

### Blood tests

Venous blood samples were collected after an overnight fasting (12 hours) for determination of total cholesterol, HDL-cholesterol, triglycerides and glucose levels. HDL-cholesterol levels were calculated by the Friedewald formula.

### Evaluation of obstructive sleep apnea

The diagnosis of OSA was determined by a home, portable monitoring device, the Watch-PAT, which indirectly detects apnea-hypopnea events by identifying sympathetic activities related to these events. After the test, results are automatically read and analyzed by a computer program.^16^ Watch-PAT provides an algorithm able to differentiate between sleep and awake state every 30 seconds, and to calculate the respiratory disturbance index (RDI) using the total sleep time rather than the total recording time at rest. The actigraphy algorithm provides an accurate measure of sleep and wake states in normal subjects and patients with OSA. This simple method for evaluation of sleep total time is a useful tool to accurately quantify OSA in the home environment.^17^

The American Academy of Sleep Medicine recognizes the Watch-PAT device as a useful alternative for the diagnosis of OSA, since it allows a manual or automatic edition of the scores obtained. Besides, there are not many technical failures with the use of the Watch-PAT at home.^13,18^ The analysis algorithm uses four functions to detect different parameters including the apnea-hypopnea index (AHI), RDI, oxygen desaturation index (ODI), the minimum, mean and maximum oxygen saturation and sleep stages.^16^

Patients were divided into two groups based on the AHI: group 1 with an AHI < 15 events/hour and group 2 with an AHI ≥ 15 events/hour, aiming to separate patients with moderate/severe OSA (group 2) from those without OSA (AHI < 5 events/hour) or mild OSA (AHI = 5-14 events/hour).

### Ambulatory blood pressure monitoring

Twenty-four-hour ABPM was performed using the Spacelabs 90207 monitor (Spacelabs Inc., Redmond, WA, USA). The cuff, with a size appropriate for the patient’s arm circumference, was placed on the non-dominant upper-arm. This monitor is validated by the British Hypertension Society and by the Brazilian Society of Cardiology.^19^ The readings were taken every 20 minutes during the day and every 30 minutes at night. During the monitoring period, subjects recorded the awake and sleep periods to calculate mean BP during these periods. Patients were instructed to avoid sleeping for more than one hour during the day. The ABPM was considered adequate if 70% of the measures were successfully obtained. The percentage of nocturnal BP decrease for systolic and diastolic pressures was calculated as the mean of diurnal BP minus the mean nocturnal BP, multiplied by 100 and divided by the mean diurnal BP. BP load was considered abnormal when more than 30% of the valid BP readings in the ABPM were above the normal limits. MH was defined as a casual BP lower than 140/90 mmHg and 24-hour BP higher than 130/80 mmHg in the ABPM, and/or awake BP greater than 135/85 mmHg and or sleep BP greater than 120/70 mmHg.^11^

### Statistical analysis

Data were analyzed by the Statistical Package for Social Sciences (SPSS) version 18.0 (SPSS Inc., Chicago, IL, USA), and the results expressed as mean ± standard deviation. Continuous variable showed a normal distribution according to the Kolmogorov-Smirnov test. The unpaired Student's t test was used to compare the mean between the groups. Categorical variables were compared using the chi-square test (χ^2^) and expressed as percentage of frequency distribution. Correlations were assessed by the Pearson correlation test. Sample size was estimated based on previous studies on 24-hour systolic BP in similar populations.^9^ Assuming a level of significance of 5% and a standard deviation of 8 mmHg, 23 patients in each group would have a power of 80% to detect a difference of 5 mmHg in 24-hour systolic BP between the groups.

## Results

A total of 86 participants were selected. However, 3 patients had arterial hypertension during the first visit, and 2 subjects refused to perform the ABPM. Therefore, full examination was performed in 81 patients. Mean age was 42 ± 6 years, and mean BMI was 33.8 ± 3.0 kg/m^2^. Group 1 was composed of 55 individuals (68%), and group 2 was composed of 26 (32%) individuals.

**Figure 1:**
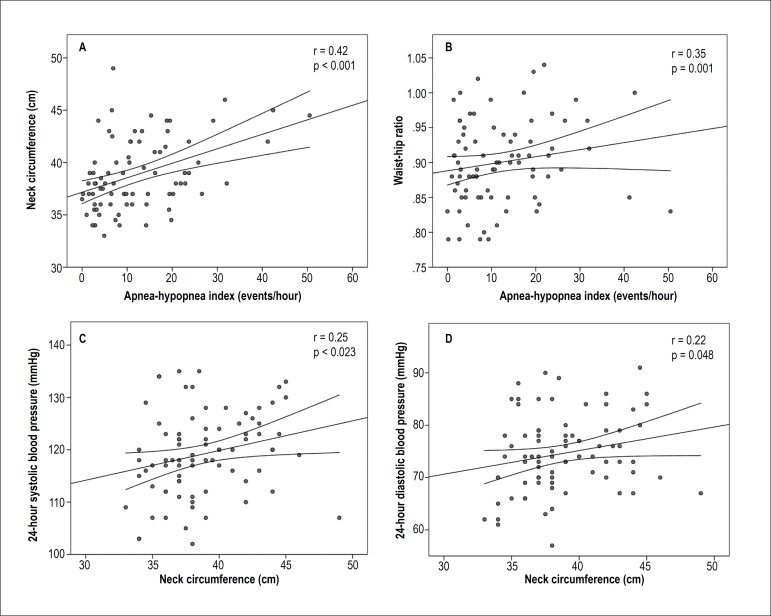
Positive correlations of apnea-hypopnea index with neck circumference (A) and waist-hip ratio (B), and of neck circumference with 24-hour systolic blood pressure (C) and 24-hour diastolic blood pressure (D).

Despite similar mean age in both groups, male sex was predominant in group 2 (Table 1). Also, although group 1 and 2 had similar BMI and WC, greater NC and greater waist-hip circumference (WHC) were observed in group 2 as compared with group 1 (Table 1). There were significant, positive correlations of AHI with NC (r = 0.42, p < 0.001) and WHC (r = 0.35, p = 0.001), and of NC with 24-hour systolic BP (r = 0.25, p = 0.023) and 24-hour diastolic BP (r = 0.22, p = 0.048) (Figure 1).

**Table 1:** Anthropometric, laboratory and Watch PAT data

Variables	Group 1 (n = 55) AHI < 15 events/h	Group 2 (n = 26) IAH ≥ 15 events/h	p Value
Male sex, n (%)	10 (18.2)	12 (46.2)	0.008*
Age (years)	41 ± 7	44 ± 6	0.170
BMI (kg/m^2^)	33.8 ± 2.9	33.9 ± 3.2	0.850
Waist-hip ratio (cm)	0.89 ± 0.05	0.94 ± 0.05	0.001*
Neck circumference (cm)	38.0 ± 3.7	40.5 ± 3.2	0.002*
Waist circumference (cm)	104.3 ± 8.3	108.5 ± 7.6	0.030
Glucose (mg/dl)	87.5 ± 11.7	91.8 ± 30.3	0.375
Cholesterol Total (mg/dl)	202.7 ± 41.3	203.6 ± 39.9	0.926
LDL-cholesterol (mg/dl)	128.3 ± 35.5	127.5 ± 34.9	0.918
HDL-cholesterol (mg/dl)	50.4 ± 14.4	48.1 ± 9.5	0.453
Triglyceride (mg/dl)	119.7 ± 71.9	140.4 ± 83.6	0.257
AHI (events/h)	6.4 ± 4.1	24.4 ± 8.8	< 0.001*
RDI (events/h)	11.8 ± 5.1	28.6 ± 8.9	< 0.001*
ODI (events/h)	3.0 ± 2.4	14.5 ± 6.9	< 0.001*
Mean O_2 ‑ _saturation (%)	95.8 ± 1.2	94.3 ± 1.4	< 0.001*
REM sleep (%)	24.0 ± 7.4	26.0 ± 8.2	0.249

Data shown as mean ± standard deviation; BMI: body mass index; LDL: low-density lipoprotein; HDL: high-density lipoprotein; AHI: apnea-hypopnea index; RDI: respiratory disturbance index; ODI: oxygen desaturation index; REM: rapid eye movement. Continuous variables were analyzed by the unpaired Student t-test, and categorical variables by the chi squared test (χ^2^).

* p < 0.05.

**Figure 2:**
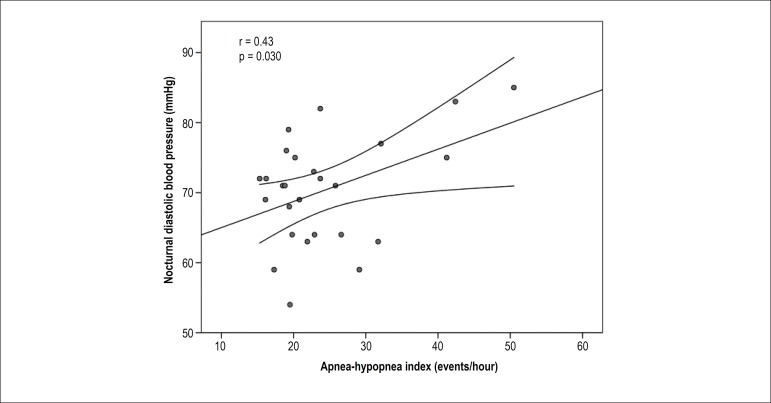
Positive correlation between apnea-hypopnea index and nocturnal diastolic blood pressure in the group of obese individuals with moderate-to-severe obstructive sleep apnea.

Systolic and diastolic casual BPs were similar between the groups. Nevertheless, group 2 had higher diurnal and nocturnal BP levels in the ABPM, and higher nocturnal diastolic BP load (Table 2). Also in group 2, nocturnal diastolic BP was positively correlated with AHI (r = 0.43, p < 0.05) (Figure 2), and the frequency of MH was slightly higher (50% vs. 33%, p= 0.103) than in group 1 (Table 2).

**Table 2:** Casual blood pressure and 24-hour ambulatory blood pressure monitoring results

Variables	Group 1 (n = 55) AHI < 15 events/h	Group 2 (n = 26) AHI ≥ 15 events/h	p Value
Casual SBP (mmHg)	121.4 ± 8.1	123.6 ± 7.7	0.321
Casual DBP (mmHg)	77.6 ± 7.6	79.1 ± 6.8	0.393
24h-SBP (mmHg)	117.6 ± 8.5	122.3 ± 6.2	0.014*
24h-DBP (mmHg)	73.1 ± 7.3	77.7 ± 6.2	0.008*
Awake SBP (mmHg)	120.5 ± 8.5	125.7 ± 6.1	0.007*
Awake DBP (mmHg)	76.1 ± 7.6	81.3 ± 5.7	0.003*
Sleep SBP (mmHg)	110.6 ± 9.9	115.3 ± 7.7	0.036*
Sleep DBP (mmHg)	65.9 ± 8.4	70.4 ± 7.7	0.025*
Dipping SBP, n (%)	19 (76)	6 (24)	0.297
Diurnal SBP load (%)	12.6 ± 16.7	16.8 ± 18.8	0.305
Diurnal DBP load (%)	21.6 ± 24.6	32.2 ± 24.7	0.074
Nocturnal SBP load (%)	22.8 ± 26.7	29.1 ± 26.2	0.322
Nocturnal DBP load (%)	31.3 ± 27.3	44.6 ± 25.9	0.041*
Nocturnal hypertension, n (%)	17 (30.9)	16 (61.5)	0.009*
Masked hypertension, n (%)	18 (33)	13 (50.0)	0.103

Data shown as mean ± standard deviation; SBP: systolic blood pressure; DBP: diastolic blood pressure. Continuous variables were analyzed by the unpaired Student's t-test, and categorical variables were compared using the chi-squared test (χ^2^).

* p < 0.05.

## Discussion

The findings of the present study showed that asymptomatic, obese subjects with moderate/severe OSA had higher 24-hour BP than subjects without OSA and/or with mild OSA. In addition, nocturnal diastolic BP was correlated with AHI. The main mechanisms of increased BP in patients with OSA are increased sympathetic activity, renin-angiotensin system dysfunction, endothelial dysfunction, hypoxemia, and disruption of normal sleep. These changes lead to increased peripheral vascular resistance and a predominantly diastolic hypertension.^8,20^

In obese individuals, the prevalence of OSA is higher than in non-obese subjects.^7^ In the present study, the percentage of patients with moderate/severe OSA was similar to those reported in previous reports.^21-23^ In the Wisconsin study, approximately 9% of men and 4% of women aged between 30 and 60 years had AHI ≥ 15 events/hour.^24^ In a similar study, approximately 13% of men and 9% of women had moderate/severe OSA (AHI ≥ 15 events/hour).^25^ In this study, we also observed a higher number of men than women in the group of moderate/severe OSA.^26^

The relationship between BMI and OSA is controversial. Although such correlation was not found in two previous studies,^27,28^ in the Sleep Heart Health Study,^6^ which evaluated 6,120 individuals in a hospital population, BMI was an independent risk factor for OSA, with an odds ratio of 1.55-1.60. In the present study, we did not find an association between BMI and OSA, which may be explained by the small sample size and narrower range of BMI for the inclusion criteria (BMI ≥ 40 kg/m^2^ was excluded). Besides, increased visceral adiposity may be more important in OSA physiopathology than overall obesity.

The anthropometric parameters NC and WHR could be used to identify individuals at high risk for OSA. In a study with 192 patients suspected of OSA, WHR was associated with moderate-to-severe OSA.^27^ Similar results were found in a study comparing individuals with different degree of snoring, indicating a significant difference in WHR between the groups.^29^

With respect to NC, a study on 129 individuals suspected of OSA reported that this anthropometric parameter was an independent risk factor for OSA.^30^ In the present study, both NC and WHR were significantly higher in patients with moderate/severe OSA, despite similar BMI and WC between the groups. This finding corroborates previous studies, suggesting that both NC and WHR could be routinely assessed in obese outpatients, to identify those patients at higher risk for OSA.

The current study also found that subjects with moderate-to-severe OSA had higher systolic and diastolic BP in the ABPM. According to cross-sectional studies on OSA, hypertension is more prevalent in OSA patients, even after controlling for confounding factors, such as age and obesity.^31^ Besides, analysis of BP circadian rhythm by the ABPM may reveal other prognostic information, such as increased prevalence of MH and increased nocturnal BP. The identification of patients with MH is important in daily clinical practice, since previous studies suggested that these patients have more target-organ injuries, including microalbuminuria and left ventricular hypertrophy.^32,33^ Furthermore, a meta-analysis of seven studies and 11,502 patients reported that MH patients have twice the risk of cardiovascular death than normal BP subjects.^34^ The prevalence of MH in the general population varies from 16 to 24%.^33^ However, in OSA patients, these values may be even higher: two previous studies reported a prevalence of nearly 30% of MH in OSA patients.^8,9^ In accordance with these studies, the present study showed a slightly higher percentage of MH in the moderate/severe OSA group.

Previous studies have suggested a relationship between OSA and nocturnal hypertension. The authors suggest that increased adrenergic activity, hypoxemia, and sleep disruption could explain the increased nocturnal hypertension in patients with OSA.^32,35^ Increased nocturnal BP may be associated with increased inflammatory markers, which may explain the increased risk for cardiovascular complications.^36^

Two studies showed a relationship between OSA and nocturnal diastolic hypertension.^37,38^ In the first study, 84% of patients with mild to moderate OSA were considered “non-dippers” (without nocturnal dipping).^11^ In the second study, the authors observed nocturnal arterial hypertension and absence of nocturnal BP dipping in OSA patients, in addition to a significant correlation between nocturnal arterial hypertension, absence of nocturnal dipping and AHI.^39^ In our study, higher values of BP in the ABPM, and increased nocturnal diastolic pressure load were found in patients with moderate/severe OSA. Also, there was a correlation between nocturnal diastolic BP and AHI. However, no significant difference in nocturnal dipping was found between the groups.

This study has some limitations. First, OSA was diagnosed by a portable monitor rather than polissonography, which is the gold-standard diagnostic method. Nevertheless, studies on validation of the Watch-PAT equipment showed similar results between measures taken by this system and those obtained by polissonography.^17,18,40^ Second, the higher prevalence of men in the group with higher AHI may have influenced the anthropometric results. Third, the cross sectional design of the study does not allow us to conclude a causal relationship between OSA and arterial hypertension. However, the use of ABPM permitted the measurements of nocturnal BP, and the identification of MH and nocturnal arterial hypertension. 

## Conclusions

Asymptomatic obese individuals with moderate/severe OSA have higher 24-hour systolic and diastolic BP in comparison with those with absent/mild OSA, despite normal casual BP. These results indicate that the ABPM may be useful in the assessment of asymptomatic obese patients with moderate-to-severe OSA. Prospective studies are needed to confirm this hypothesis.
